# Mad Honey Intoxication: A Case Series of 21 Patients

**DOI:** 10.5402/2011/526426

**Published:** 2011-10-26

**Authors:** Hasan Demir, Arzu Denizbasi, Ozge Onur

**Affiliations:** ^1^Department of Emergency Medicine, Fatih Sultan Mehmet Research and Training Hospital, Bostancı, 34752 Istanbul, Turkey; ^2^Department of Emergency Medicine, Marmara University Medical Faculty, Haydarpasa, 34668 Istanbul, Turkey

## Abstract

*Background*. The “grayanotoxin (mad honey)” poisoning is not known commonly, there are some case series and case reports in the medical literature about it, especially in Turkey. The aim of this study was to describe the presentation of 21 natural honey intoxication cases and to review the literature. *Material and Method*. This study is retrospective analysis of twenty one patients who were admitted to the emergency department due to honey poisoning. *Results*. Median age of 21 patients was 55. The mean length of delay after consumption is 3.4 hrs. Dizziness, weakness, excessive perspiration, nausea-vomiting, and low blood pressure were the most observed symptoms. Mean pulse rate was 56/min. Mean systolic blood pressure was 102 mmHg. The mean length of hospital stay is 14.7 hrs. Patient rhytms on arrival were as follows: 10 patients were in normal sinus rhytm, 7 sinus bradycardia, 3 nodal rhytm, 1 atrial fibrillation. Atropine was given to 18 patients. None of our patients died and all were discharged home without any complication. *Discussion*. In the emergency setting, poisoning is a clinical state which is very hard to identify. We have to keep in mind that drugs and toxins may cause lethal dysrhythmias.

## 1. Introduction

Several plants of the Ericaceae (Rhododendron) family produce grayanotoxins which can poison humans. Grayanotoxins are known to occur in honey produced from the nectar of Rhododendrons [1, 2]. There are 18 forms of grayanotoxins [3]. Grayanotoxins such as grayanotoxin I–IV are a unique class of toxic diterpenoids which are polyhydroxylated cyclic hydrocarbons that do not contain nitrogen. The principle toxic isomer in Rhododendron is grayanotoxin III. 

The grayanotoxins are neurotoxins interfering with the transmission of the action potential by blocking sodium channels in cell membranes [3]. The best-known of these intoxications involves the eating of “mad honey” contaminated by nectar with grayanotoxins. It is still one of the common food intoxications encountered for humans and livestock in Turkey. 

It causes a sharp burning sensation in the throat and is thus also referred to as bitter honey [4]. Honey produced in springtime is more toxic and sometimes contains higher concentrations of grayanotoxin than that produced in other seasons. Mad honey is used as an alternative medicine for the treatment of gastric pains, bowel disorders, and hypertension, and it is also believed to be a sexual stimulant. 

Although the “grayanotoxin (mad honey)” poisoning is not known commonly, there are some case series and case reports in the medical literature about it, especially in Turkey.

The aim of this study was to describe the presentation of 21 natural honey intoxication cases and to review the literature. 

## 2. Material and Methods

This study is retrospective analysis of twenty-one (21) patients who were admitted to the emergency department due to honey poisoning between April and June 2010. 

All of these patients were admitted to the Emergency Medicine Department. The demographic data, clinical symptoms, length of delay after consumption, length of stay in hospital, vital signs, ECG pathologies, and treatments administered to mad honey patients were noted. We enrolled the patients whose other causes (cardiac, neurologic, metabolic) were ruled out as alternative diagnoses of clinical presentation. Also the diagnosis of mad honey poisoning was based on a history of ingestion of unprocessed locally obtained honey and typical signs of dizziness, ataxia, bradydysrhythmias, diaphoresis, and hypotension. The confirmation of mad honey intoxication was mainly based on the clinical symptoms and the patients confirmed to have used the mad honey.

### 2.1. Statistical Analysis

IBM SPSS Statistics 15 (SPSS Inc., Chicago, IL, USA) is used for statistical calculation processes. The results are given as mean within 95% Confidence Intervals.

## 3. Results

There were 21 patients (13 male and 8 female) admitted to the emergency service due to the honey poisoning. Median age of patients was 55 (45–72). The mean length of delay after consumption is 3.4 hrs (95% CI; 1.5–6.2 hrs). Dizziness, weakness, excessive perspiration, nausea-vomiting, and low blood pressure were the most observed symptoms (Table 1). Mean pulse rate was 56/min (95% CI; 44–70/min). Mean systolic blood pressure was 102 mmHg (95% CI; 78–125 mmHg). 

**Figure 1:**
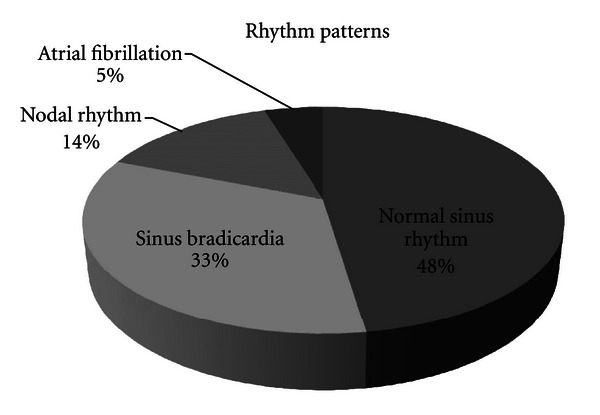
ECG rhythms of 21 patients.

The mean length of hospital stay is 14.7 hrs (95% CI; 6.3–21.2 hrs). Patient rhythms on arrival were as follows (Figure 1): 10 patients were in normal sinus rhytm, 7 sinus bradycardia, 3 nodal rhytm, and 1 atrial fibrillation. Atropine was given to 18 patients. Three of the patients did not require atropine. None of our patients died and all were discharged home without any complication.

## 4. Discussion

Mad honey poisoning is common in the eastern Black Sea region of Turkey [5]. The grayanotoxins are neurotoxins interfering with the transmission of the action potential by blocking sodium channels in cell membranes. These compounds prevent inactivation; thus, excitable cells (nerves and muscles) are maintained in a state of depolarization, during which entry of calcium into the cells may be facilitated. All of the observed responses of skeletal and heart muscles, nerves, and the central nervous system are related to the membrane effects [4]. 

The toxic effects of honey poisoning are rarely fatal and generally last for no more than 24 hours. Symptoms of poisoning occur after a dose-dependent latent period of a few minutes to 2 or more hours. In our case series, the mean length of delay after consumption is 3.4 hrs. Mad honey intoxication's symptoms are dose-related. In mild forms, dizziness, weakness, excessive perspiration, hypersalivation, nausea, vomiting, and paresthesias are present, and close followup is enough. However, severe intoxication may lead to life threatening cardiac complications such as complete atrioventricular block. In our case series, and in those of patients previously described, symptomatic sinus bradycardia with hypotension is the most frequently reported mad-honey-induced cardiac dysrhythmia [6]. All of our patients responded to intravenous fluid therapy and parenteral atropine. Overall, all of our patients reached complete recovery.

Locally produced honey is widely consumed in the Black Sea region [7]. In general, beekeepers sell their own unprocessed honey in local and regional markets. This honey is natural, unprocessed, and unregulated product. They reach the consumer directly, with no intermediate processing. In general, the severity of the honey poisoning depends on the amount ingested. But there is no standard amount of toxin in 1 g. The concentration of grayanotoxin ingested may differ greatly from case to case. Yilmaz et al. reported that the amount of honey causing poisoning is between 5 and 30 g [8]. As grayanotoxins are metabolized and excreted rapidly, patients generally regain consciousness and feel better within hours, and heart rate and blood pressure usually return to normal within 2–9 hours. Symptoms of poisoning occur after a dose-dependent latent period of a few minutes to two or more hours. In untreated cases of severe intoxication, the worst signs and symptoms last about 24 hours. 

## 5. Conclusion

Cardiac rhythm problems are one of the major challenges that we have to face. However, the therapy of the patient should be planned after taking a careful history. In the emergency setting, poisoning is a clinical state which is very hard to identify. We have to keep in mind that drugs and toxins may cause lethal dysrhythmias.

## Figures and Tables

**Table 1:** Clinical symptoms of 21 patients

Symptoms of the patient	Number of symptoms in 21 patients (% of total)
Dizziness	21 (100%)
Weakness	21 (100%)
Excessive perspiration	18 (85%)
Nausea/vomiting	18 (85%)
Low blood pressure	14 (66%)
Pre-syncope	12 (57%)
Syncope	5 (23%)
